# Loop-mediated isothermal amplification: a rapid molecular technique for early diagnosis of *Pseudomonas syringae pv. syringae* of stone fruits

**DOI:** 10.1186/s43141-020-00062-6

**Published:** 2020-10-02

**Authors:** R. Goudarzi, M. M. Mortazavi

**Affiliations:** 1 Department of Agriculture, Damghan Islamic Azad University, Damghan, Iran; 2 Stem Cell Research Center, Golestan University of Medical Sciences, Gorgan, Iran; 3 Ehya Bone Company, Growth Center, Golestan University of Medical Sciences, Gorgan, Iran; 4 Transmission Electron Microscope Lab, Biomedical Technology Wing, SCTIMSTs, Trivandrum, India

**Keywords:** SyrD, King’s B medium, PCR, LAMP, Molecular diagnosis, Sensitivity, Specificity

## Abstract

**Background:**

Pathogenic bacteria cause significant economic damages in agriculture. The detection of such bacteria is considered as a continual interest for plant pathologists to prevent disease dissemination. *Pseudomonas syringae pv. syringae* is one of the most important bacterial pathogens infecting yield and quality of stone fruits throughout the world. Biochemical assays such as a LOPAT and GATTa are common methods to detect this pathogen. Serological tests and culturing on King’s B selective medium also used to isolate this bacterium. Selective media is composed of specific and effective ingredients to inhibit the growth of certain species of microbes in a mixed culture while allowing others to grow. These are used for the growth of only selected microorganisms. King’s B medium can be used as a general medium for the non-selective isolation cultivation and pigment production of *Pseudomonas* species from foods, cosmetic samples, plants, etc.

Nevertheless, the mentioned methods are not enough accurate to differentiate the strains. On the other hand, PCR-based techniques are sensitive and efficient in detecting plant diseases. However, these techniques are not practicable for those researchers who do not have access to a thermal cycler. We have used loop-mediated isothermal amplification to couple with a target. The amplification of syrD gene using loop and bumper primers can be used to prevent disease dissemination.

**Results:**

The outcome of this investigation indicated more sensitivity of LAMP in comparison to PCR. The direct addition of SYBR Gold in microtube is more sensitive than gel in both LAMP and PCR byproducts so we can eliminate gel electrophoresis, while the LAMP showed high sensitivity and high specificity in comparison to results obtained by cultivation. The described molecular test could detect *Pseudomonas syringae pv. syringae* type in nearly 1 h, and this is the first time that Lamp molecular detection of *Pseudomonas syringae pv. syringae* particularly on stone fruits is described and introduced.

**Conclusions:**

The obtained data confirmed that LAMP is a fast, cheap, and high specific method for the rapid detection of *Pseudomonas syringae pv. syringae* to the comparison of PCR and culture.

## Background

*Pseudomonas syringae pv. syringae* is a bacterial pathogen responsible for twig, diebacks, blossom, leaf or kernel blights, leaf spots [1], and especially bacterial canker, a plant disease characterized by sunken patches of dead bark and small holes in leaves [2–4]. It can lead to diseases in more than 180 plant species such as fruit trees and annual and perennial plants [5, 6]. *Psy* damages are determined by on growing region of stone fruits and host plants [1, 7]. *Psy* is the most economically important pathogen with many pathovars in 14 species of plant pathogenic *Pseudomonas* [1].

Isolating these deleterious populations seems important, although screening isolates is considered as a laborious process because of the considerable genotypic and phenotypic diversity demonstrated by this group of bacteria [8]. Although the culture method broadly used to isolate supposed *P. syringae* strains is still presumed a sensitive technique, it causes biases related to the use of phenotypic properties. On the other hand, pathovars from the *P. syringae* group represent considerable diversity in virulence gene repertoires that cannot be used to detect the whole *P. syringae* pathovars [9]. *P. marginalis*, *P. savastanoi*, and *P. syringae* among fluorescent *Pseudomonas* species have several pathovars that are characterized based on biochemical properties and pathogenicity to host plant species [1, 7].

Morphological properties and biochemical assays (e.g., LOPAT and GATTa) [10], serologic tests [11], fatty acid profiling [12], genomic and plasmid DNA analysis [13], and protein analysis [14] are currently used for the detection and identification of *Psy* and as powerful tools for detection of numerous pathogens besides [15]. Nevertheless, the mentioned methods are not enough accurate to differentiate the strains and pathovars [10]. Pathovars of *Pseudomonas* which cultivated on KB is usually fluorescent when subjected to ultraviolet light after 24–48 h of incubation [1, 16].

Though *Psy* grows on KB medium and produces green fluorescent pigment, other bacteria belonging to *P. syringae* show positive responses to this non-specific experiment. Hence, this test could not differentiate this pathovar from other pathovars of *Pseudomonas* [10]. PCR-based techniques are sensitive and efficient in detecting plant diseases. The PCR method has been used to detect genes that participated in the production of coronatine (*CFL*), secretion of syringomycin (*syrD*), and syringomycin synthesis (*syrB*) [4].

Rep-PCR has an essential role to analyze the diversity of the pathogen leading to several bacterial diseases of stone fruits and pathovars of *P. syringae* group [17, 18]. Gasic and colleagues could detect toxin-producing genes, *syrB*, and *syrD* in *Psy* within stone fruits by Rep-PCR [19]. Kaluzna et al. identified *Pseudomonas syringae* pathovars from stone fruit trees using PCR [20]. Therefore, molecular methods must be used for the differentiation of strains [21, 22]. Loop-mediated isothermal amplification (LAMP), as a leading technology uses a heat-resistant strand-displacement DNA polymerase and 4–6 primers targeting definite DNA regions with designed secondary structures formerly [23].

The current study indicates not only loop-mediated isothermal amplification of DNA does not require thermal cycler (unlike PCR) but also can be a valid technique for the detection of Psy with higher sensitivity and specificity. In this method, syrD gene amplification is carried out by Bst DNA Polymerase at a single temperature (60 °C) using loop and bumper primers. *SyrD* is a conserved pathogenic gene involved in the secretion of the toxin syringomycin in *Psy* [24]. In 1999, *syrD* gene detection was done by the Bultreys and Gheysen’s method [4]. As syrD conserved among *Pseudomonas syringae* pathovars, the selection of gene-based LAMP and PCR tests were reasonable [25]. In this research, the identification of the putative gene in *Psy* by three methods of cultivation, PCR, and LAMP is compared.

## Methods

### Bacterial strains collection

Fifty bacterial canker samples taken from the stem, buds, twigs, and shoots were collected from Azadshahr (Golestan Province, Northern Iran) gardens of stone fruits (peach trees, *Prunus persica*). Infected samples were stored in plastic bags and restored at 4 °C. Then, a total of 50 bacteria were isolated from 50 infected parts of peach trees.

### Bacterial culture

Fifty samples were divided into two groups: one cultured on selective King’s B medium for detection of the strains (Fig. 8). For this purpose, all samples are kept in nutrient broth containing 20% glycerol at − 85 °C and cultured on KB at 25 °C for 48 h before usage [16]. After 24–48 h of incubation, fluorescence on KB is observed under UV light [1]. Another group used for genomic DNA extraction carried out using Bioron Ron’s Plant DNA Mini Kit (Bioron, Germany).

### Genomic DNA extraction

Genomic DNA was extracted by using Ron’s Plant DNA Mini Kit (Bioron, Germany).

### SyrD primer pair designing for PCR

The primer design for the specific identification of putative pathovars of the *P. syringae* group is needed to target distinct and well-defined regions of the genome. *SyrD* sequence, a 446 bp conserved sequence found in the *Psy* genome [26], was used as a template for primer designing. Primers were analyzed using the NCBI primer blast online tool (https://www.ncbi.nlm.nih.gov/tools/primer-blast/) for specificity. The sequence of PCR primer pair was shown in Table 1 [27].

**Table 1:** Sequences of PCR primers for amplification of the syrD DNA from Psy

Primer	Length (bp)	Sequence
F	21	5′AAACCAAGCAAGAGAAGAAGG3′
R	21	5′GGCAATACCGAACAGGAACAC3′

### PCR reaction

To amplify the syrD conserved domain gene, PCR was carried out in total 25 μl reaction volume containing 12.5 μl AMP fast PCR Master Mix (Takara, Japan), 10.5 μl H_2_O, 0.5 μl each F and R primers, and 1 μl (200 ng) of genomic DNA. PCR was carried out for 30 cycles at beneath condition: 1 min initial denaturation at 94 °C, 5 min denaturation at 98 °C, 5 s primer annealing at 55 °C, and 10 s elongation at 72 °C [28]. PCR amplification reactions were done in a C1000 Touch™ Thermal Cycler (Bio-Rad, USA) and stained with 1% agarose gel, and SYBR Gold 1 kb molecular weight ladder was used.

### SyrD primer pairs designing for LAMP

**Fig. 1:**
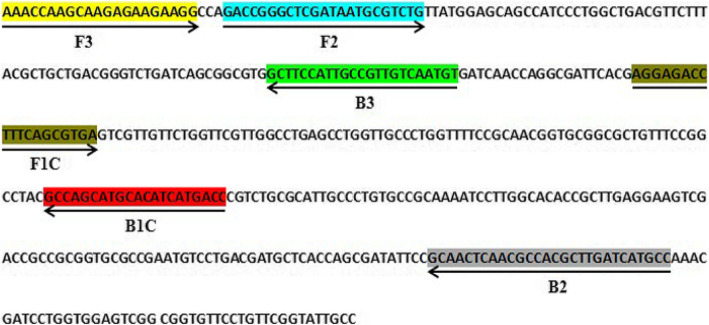
Location and partial sequence of loop-mediated isothermal amplification (LAMP) primer set targeting putative syrD sequence of Pseudomonas syringae.pv syringae specific DNA. Locations for two outer (F3 and B3), two inner (FIP [F1c-F2], and BIP [B1c-B2]) primers are indicated in the figure by colors. FIP is a hybrid primer consisting of the F1c sequence and the F2 sequence, and BIP is a hybrid primer consisting of the B1c sequence and the B2 sequence. Arrows indicate the extension direction

*SyrD-like* conserved domain (Gene Bank accession no. KC999805.1) in toxin-producing strains were used for LAMP primer design. The designed primers were synthesized by Bioneer Ltd (South Korea). These primers were synthesized using Primer 3 software. Two primer pairs were checked by NCBI Primer-BLAST online tool (https://www.ncbi.nlm.nih.gov/tools/primer-blast/) to ensure that it is specific for the microorganism. One pair of primers was given from the PCR method. The sequences of the LAMP primer pairs were shown in Table 2. The schematic diagram of the LAMP primer design and detailed locations of primers in the target DNA sequences are shown in (Fig. 1).

**Table 2:** Sequences of LAMP primers for amplification of the syrD DNA from Psy

Primer	Length (bp)	Sequence
F3	21	5′AAACCAAGCAAGAGAAGAAGG3′
B3	21	5′GGCAATACCGAACAGGAACAC3′
FIB	46	5′CAGGGATGGCTGCTCCATAACCAGACCGGGCTCGATAATGCGTCTG3′
BIP	51	5′GCAACTCAACGCCACGCTTGATCATGCGCCGACTCCACCAGGATCGTTTGG3′

### LAMP primer amplification

The concentration of LAMP was carried out in a total 20 μl reaction volume containing FIP, BIP, F3 and B3 primers (0.8 μM each), 1.4 mM dNTPs (Fermentas), 0.8 M betaine (Sigma), 8 mM MgSO4 (Sigma), 8 units of the Bst DNA polymerase large fragment (8000 U, New England Biolabs), 1 ng of target DNA, and 9.92 μl of distilled water. In LAMP, the large fragment of Bst DNA polymerase with strand-displacement activity employs loop and bumper primers for DNA fabrications. The mixture was incubated at 60 °C for 1 h. LAMP products were further observed on 1% agarose gel for staining with SYBR Gold [23]. A 1 kb molecular weight ladder was used.

### Gel staining of PCR and LAMP products

**Fig. 2:**
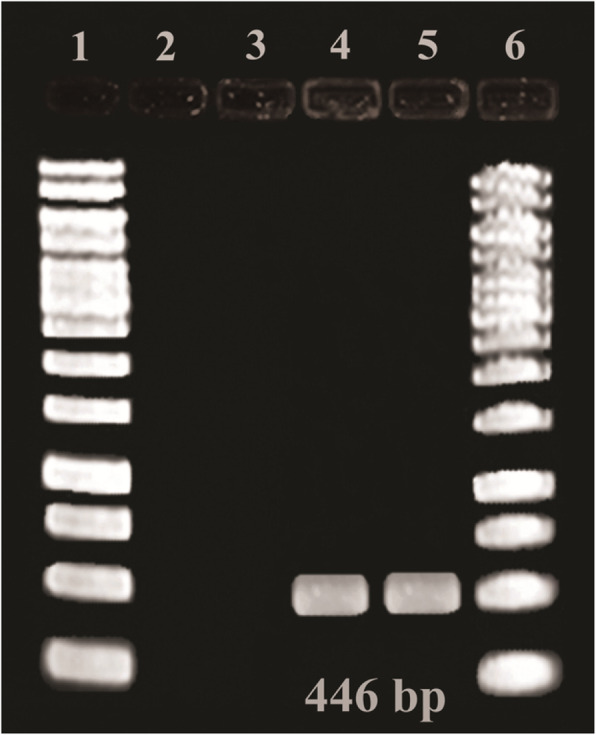
The results of *syrD* gene PCR amplification. Lanes 1 and 6, 1kB DNA ladder (Fermentas); lane 2, blank; lane 3, negative control (*Xanthomonas ssp*); lanes 4 and 5 show positive responses. A 1 kb molecular weight ladder was used, and all products were stained with SYBR Gold

**Fig. 3:**
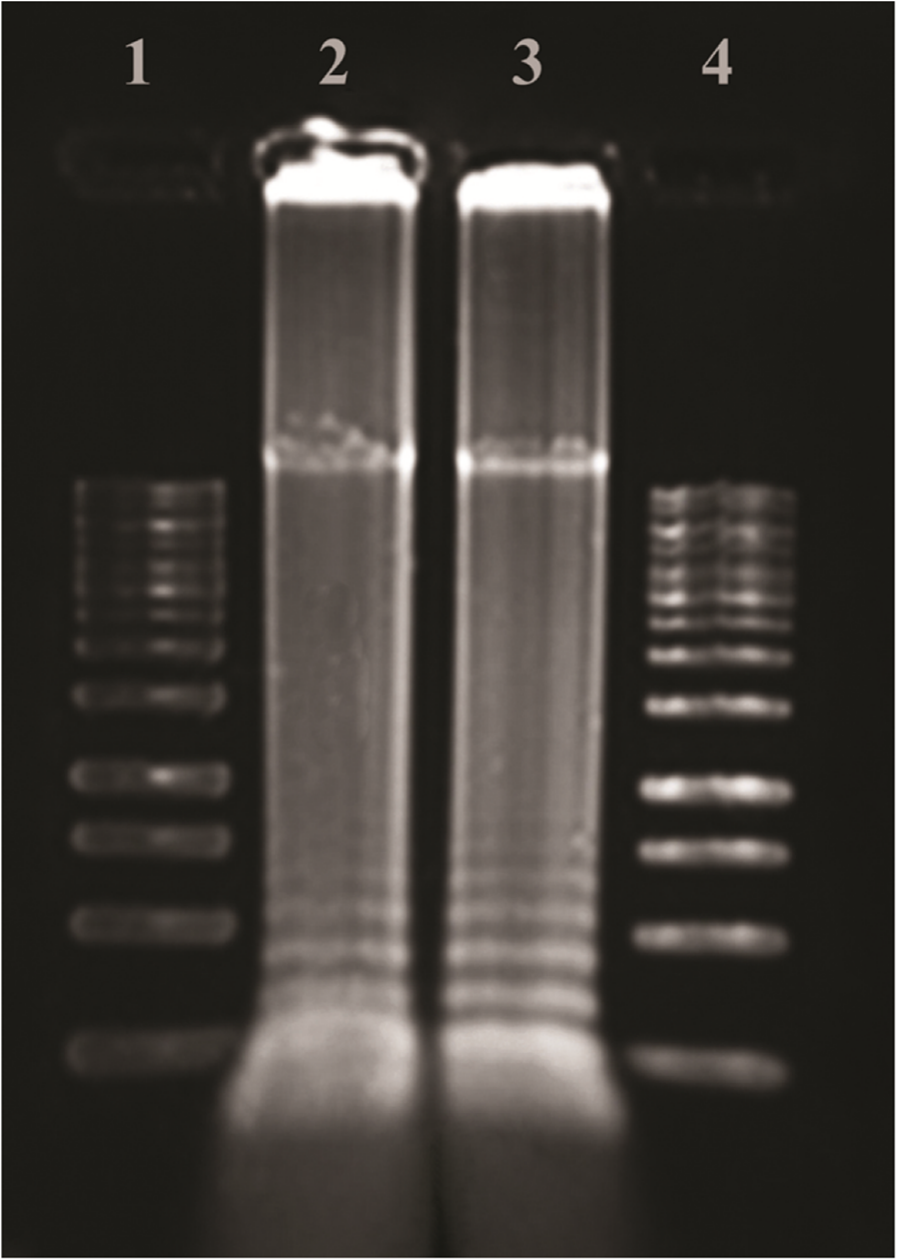
The results of the *syrD* gene LAMP. Lanes 1 and 4, 1 kB DNA ladder (Fermentas); lanes 2 and 3 show LAMP results of *Pseudomonas syringae pv. syringae* with two pairs of primers

**Fig. 4:**
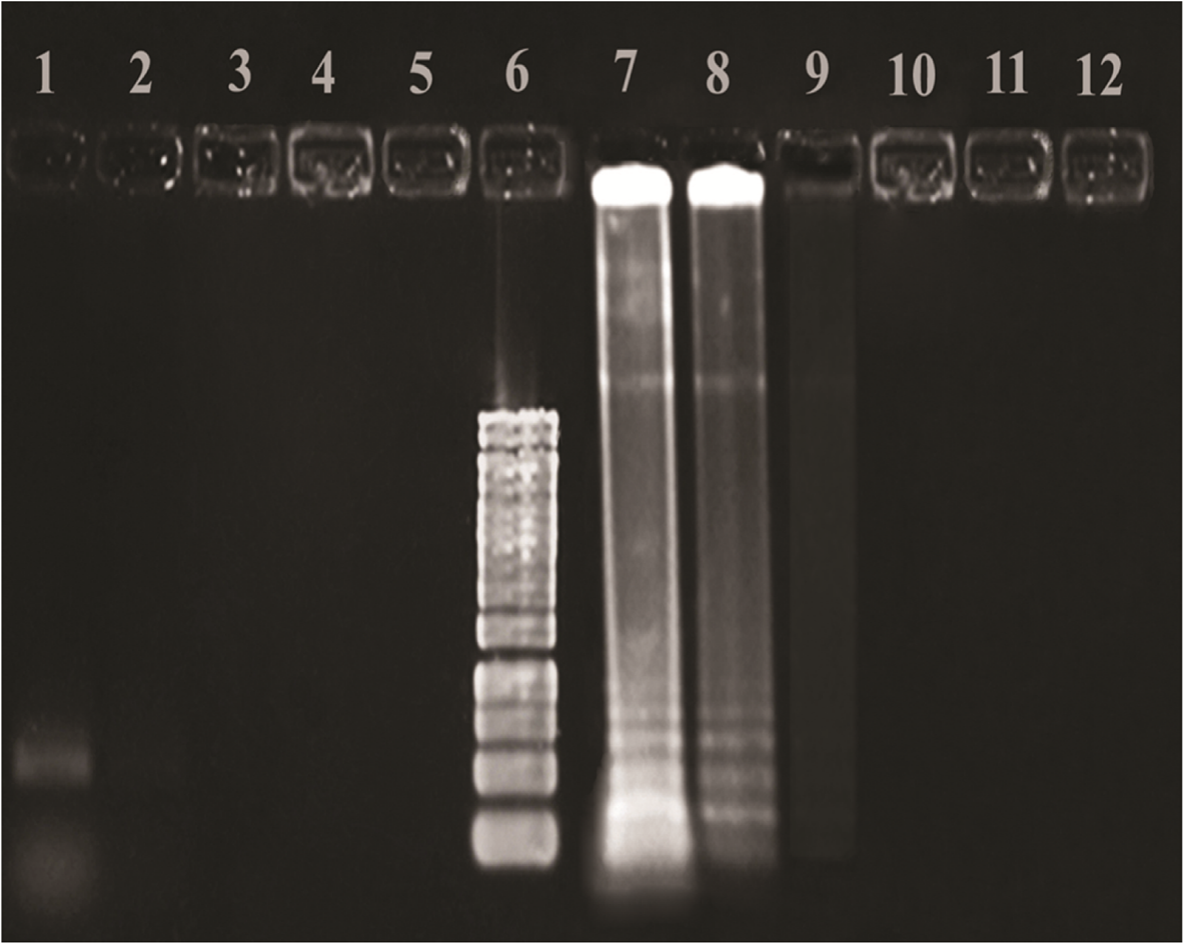
The comparison of electrophoresis-based methods of PCR and LAMP products in terms of sensitivity. Lane 1, PCR product without dilution 10 (200 ng/μl); lane 2, PCR product diluted by 10^− 1^ (20 ng/μl); lane 3, PCR product diluted by 10^− 3^ (2 ng/μl); lane 4, PCR product diluted by 10^− 3^ (0.2 ng/μl); lane 5, PCR product diluted by 10^− 4^ (0.02 ng/μl); and lane 6, 1 kb DNA ladder. Lane 7, LAMP product without dilution 10 (1 ng/μl); lane 8, LAMP product diluted by 10^− 1^ (0.1 ng/μl); lane 9, LAMP product diluted by 10^− 2^ (0.01 ng/μl); lane 10, LAMP product diluted by 10^− 3^ (0.001 ng/μl); lane 11, LAMP product diluted by 10^− 4^ (0.0001 ng/μl); and lane 12, LAMP product diluted by 10^− 5^ (0.00001 ng/μl)

The amplified PCR products were stained by SYBR Gold on agarose gel [29] (Fig. 2). LAMP uniquely amplifies DNA for producing DNA amplicons with ladder shape behaviors in gel electrophoresis [23] (Fig. 3). Equal dilutions were prepared for both LAMP and PCR products and run on electrophoresis gel for sensitivity comparison of PCR and LAMP products (Fig. 4).

### Direct visualization of PCR and LAMP products by SYBR Gold

**Fig. 5:**
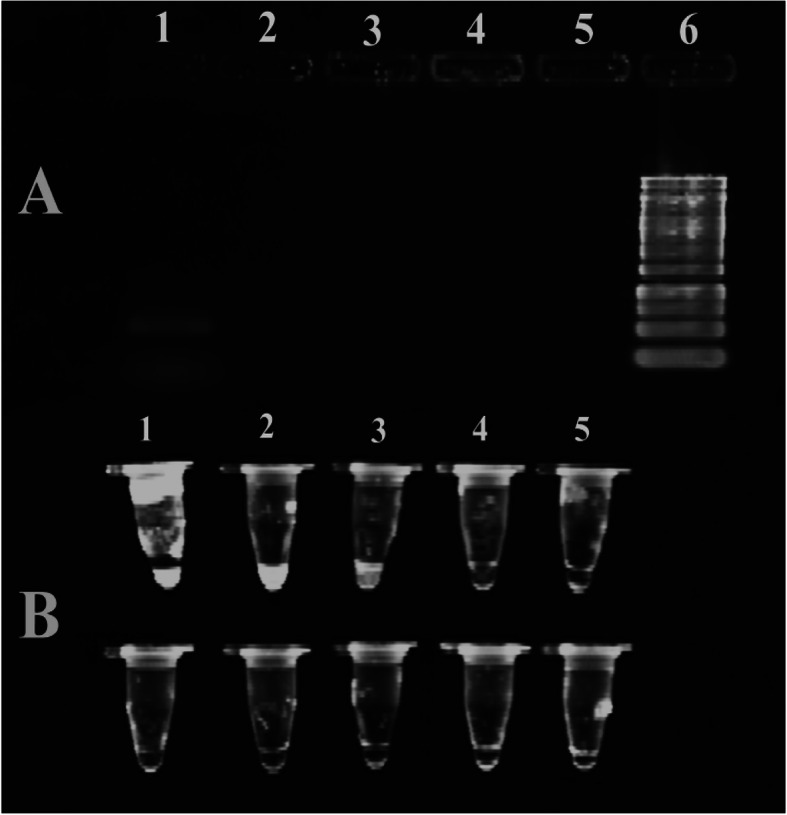
The comparison of gel-free and gel-based SYBER Gold staining: the upper panel. Part A: lane 1, PCR product without dilution 10 (200 ng/μl); lane 2, PCR product diluted by10^− 1^ (20 ng/μl); lane 3, PCR product diluted by 10^− 2^ (2 ng/μl); lane 4, PCR product diluted by 10^− 3^ (0.2 ng/μl); lane 5, PCR product diluted by 10^− 4^ (0.02 ng/μl); and lane 6, 1 kb DNA ladder. Part B: the upper row. Sample 1, a mixture of 10 μl PCR product (X) and 10 μl SYBR Gold. Sample 2, the mixture of 10 μl X/10 PCR product and SYBR Gold. Sample 3, the mixture of 10 μl X/10 PCR product and SYBR Gold. Sample 4, 10 μl X/10000 PCR product and SYBR Gold. Sample 5, 10 μl of X100000 PCR product and SYBR Gold. The lower row, 20 μl SYBR Gold

**Fig. 6:**
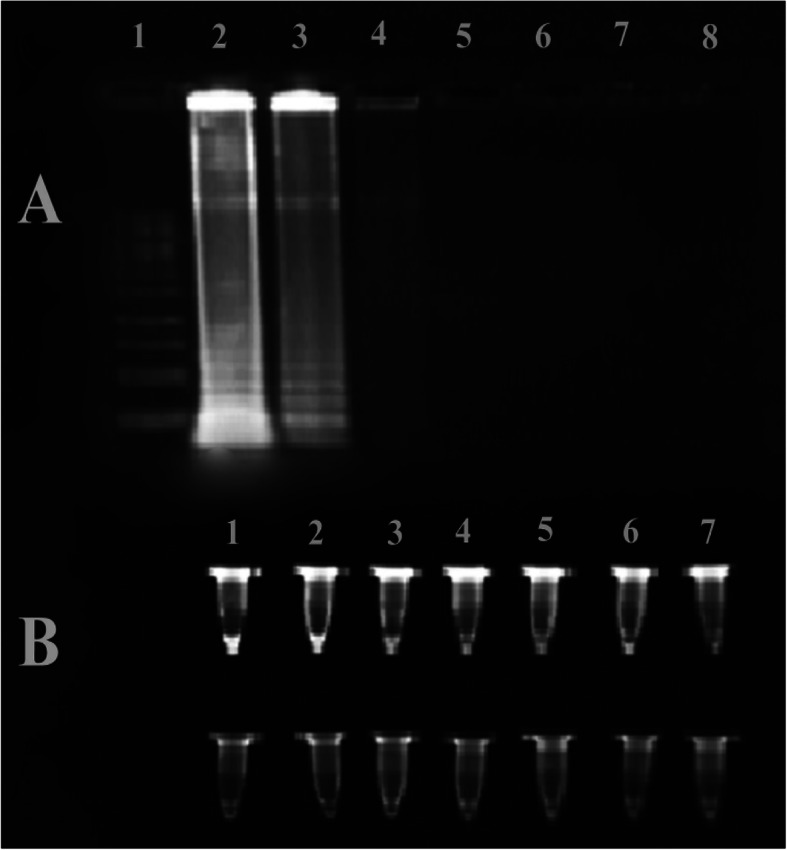
The comparison of gel-free and gel-based SYBER Gold staining: the upper panel. Part A: lane 1, 1 kb DNA ladder; lane 2, LAMP product without dilution 10 (1 ng/μl); lane 3, LAMP product diluted by 10^− 1^ (0.1 ng/μl); lane 4, LAMP product diluted by 10^− 2^ (0.01 ng/μl); lane 5, LAMP product diluted by 10^− 3^ (0.001 ng/μl); lane 6, LAMP product diluted by 10^− 4^ (0.0001 ng/μl); and lane 7, LAMP product diluted by 10^− 5^ (0.00001 ng/μl). Part B: the upper row. Sample 1, a mixture of 10 μl LAMP product (X) and 10 μl SYBR Gold. Sample 2, a mixture of 10 μl 1/10 LAMP product and SYBR Gold 3. A mixture of 10 μl 1/100 LAMP product and SYBR Gold. Sample 3: a mixture of 10 μl 1/1000 LAMP product and SYBR Gold. Sample 4, 10 μl 1/10000 LAMP product and SYBR Gold. Sample 5, 10 μl of 1/100000 LAMP product. Sample 6, 10 μl of 1/1000000 LAMP product and SYBR Gold. Sample 7, 10 μl of 1/1000000 LAMP product. Part B: the lower row, 20 μl SYBR Gold

To remove electrophoresis step optionally, SYBR Gold was directly added to the PCR and LAMP products in the microtube to be visualized by UV transilluminator (Figs. 5 and 6) [30, 31].

### LAMP analysis with different microorganisms

**Fig. 7:**
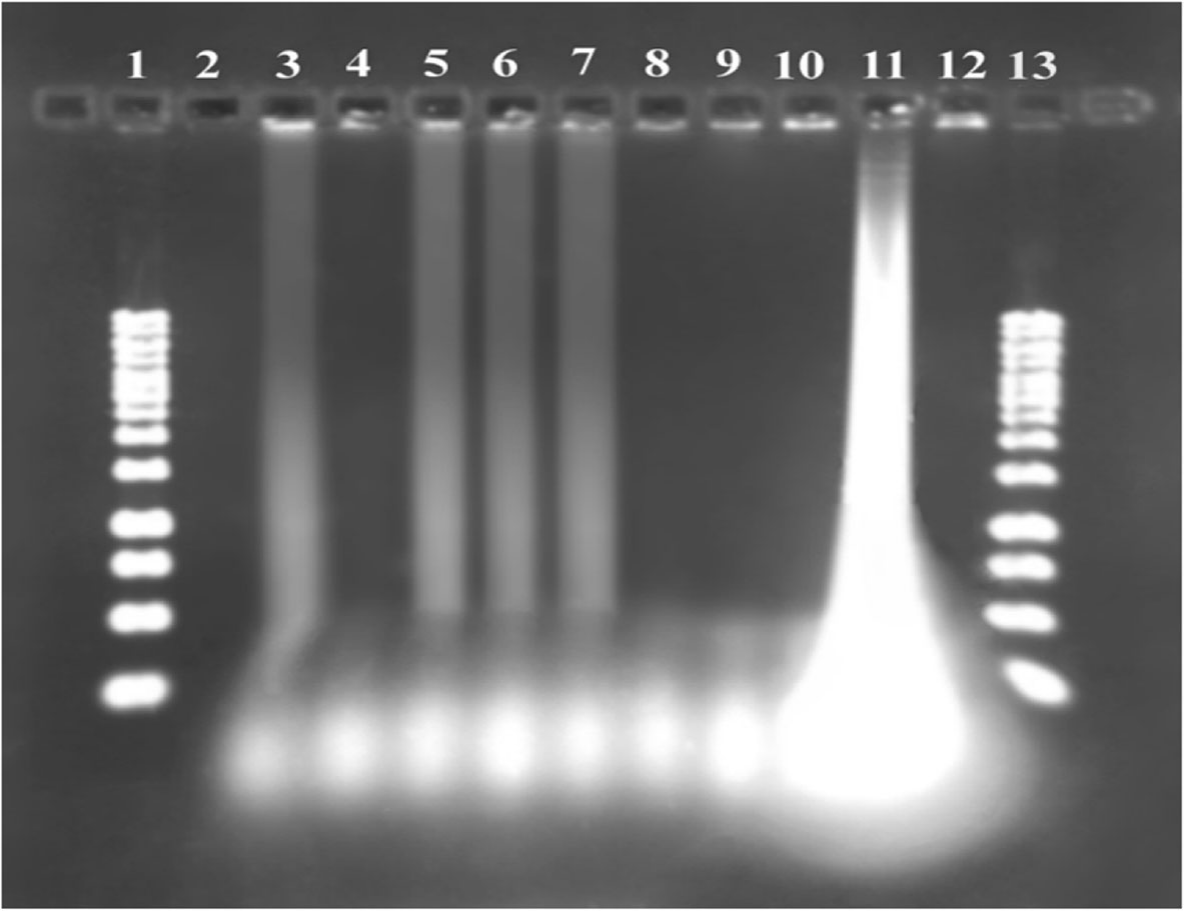
LAMP process on different microorganisms in terms of specificity of the designed primers that only detect stone fruits: lanes 1 and 13, 1 kb DNA ladder; lane 2, blank; lane 3, cherry, lane 4, tomato; lane 5, apricot; lane 6, almond; lane 7, peach; lane 8, olive; lanes 9 and 10, positive controls (*Xanthomonas campestris* ssp); lane 11, plum; and lane 12, negative control (*Brenneria* spp)

To determine that the primers are only specified for the stone fruits, the LAMP process was carried out on 10 different microorganisms. The results of these analyses are shown in Fig. 7.

## Results

### Conventional PCR and LAMP detection of syrD gene

The conventional PCR amplification on syrD using outer primers F and R was used to verify whether the correct target was amplified and an expected 446 bp fragment was obtained (Fig. 2).

Two sets of primers were designed for the *Pseudomonas syringae pv. syringae* amplification. To examine whether these sets of primers were able to amplify their target genes, LAMP reactions were conducted and analyzed by agarose gel electrophoresis are shown in (Fig. 3).

### The comparison of PCR and LAMP products in terms of sensitivity

To determine the LOD of the LAMP and PCR assay validation, Two sets of serial dilution of *Pseudomonas syringae pv. syringae.* (10, 10^− 1^, 10^− 2^, 10^− 3^, 10^− 4^) from 200 ng genomic DNA for PCR and (10, 10^− 1^, 10^− 2^, 10^− 3^, 10^− 4^, 10^− 5^) from 1 ng of genomic DNA for LAMP were prepared. Diluted templates were amplified using conventional PCR and LAMP. Both products were detected by gel electrophoresis stained with SYBR Gold (Fig. 4).

### The comparison of gel-free and electrophoresis-based methods of PCR products in terms of sensitivity

To compare the sensitivity of PCR and PCR-free techniques, the same dilutions in the two conditions were considered electrophoresis and then staining in 1X SYBR Gold, and direct mixing with SYBR Gold 1X (Fig. 5). In part A, only PCR product without the dilution is positive, but in part B, after the direct mixing of PCR products with SYBR Gold, the sensitivity is much higher. So with this method, we can eliminate gel electrophoresis and have faster detection.

### The sensitivity of gel-free and gel-based staining of LAMP byproducts

This method is as same as Fig. 5 but compares the sensitivity of LAMP and LAMP-free techniques in the same dilutions in two conditions considered: electrophoresis and then staining in 1X SYBR Gold (gel-based) and direct mixing with SYBR Gold 1X (gel-free). In part A, LAMP product without the dilution 10 (1 ng/μl) and 10^− 1^ (0.1 ng/μl) is positive, but in part B, after direct mixing of LAMP products with SYBR Gold, the 10^− 2^ (0.01 ng/μl) dilution is positive too, and the sensitivity of the directly mixed is much higher as we said before, and with this method, we can eliminate gel electrophoresis and have faster detection (Fig. 6).

### The specificity of designed primers on stone fruits

As we test only peach sample for comparison of LAMP and culture, we should be sure that the designed primers were only for *Pseudomonas syringae pv. syringae* of the stone fruit detection. Identification of these primers were tested on 10 different microorganisms showed in Fig. 7.

### Bacterial culture on King’s B medium

**Fig. 8:**
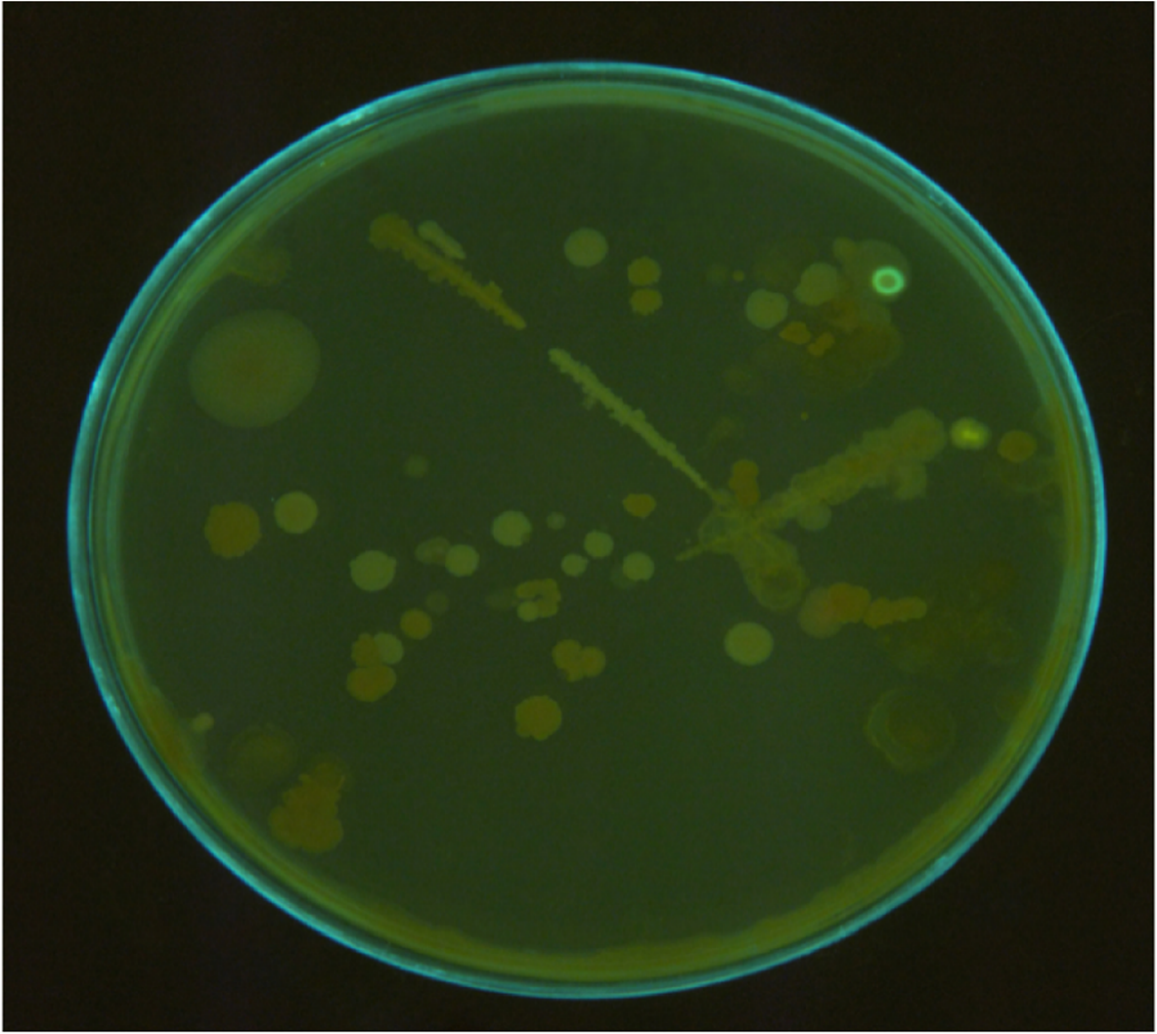
Bacterial culture on King’s B medium (1 plate of 50 samples). After 48–72 h of incubation, fluorescence on King’s medium B was observed under UV light. Fluorescence colonies showed *Pseudomonas syringae pv. syringae* has grown on this medium

To determine whether *Psy* was identified correctly, the suspected samples were cultured on King’s B medium at 28 °C. The results indicate that the bacterium has been identified correctly (Fig. 8). After 48–72 h of incubation, fluorescence on King’s medium B was observed under ultraviolet light. Fifteen *Pseudomonas syringae* strains from 50 samples fluoresced on the KB medium.

### Statistical analysis for the comparison of LAMP and culture

To compare the degree of the specificity and sensitivity of the LAMP process and standard culture, all 50 samples were cultured on King’s B medium and the LAMP process was carried out on 50 samples of *Psy*. The overall results are presented in Table 3. The results confirmed the high sensitivity and specificity of LAMP assays in comparison to direct culture.

**Table 3:** The comparison of LAMP assays with direct bacterial cultivations

Tests and results		Culture		Sensitivity	Specificity
		Pos	Neg		
LAMP	Pos	15	0	100%	100%
	Neg	0	35		

Statistical analysis includes true positives (TP) and true negatives (TN) which were determined by bacterial culture results, with false positives (FP) and false negatives (FN) attributed to findings from the LAMP assays: Sensitivity = TP/TP + FN; Specificity = TN/TN + FP [32].

## Discussion

The current study showed that isothermal amplification of *syrD* gene using PCR and LAMP primers along with bacterial cultivation on King’s B medium can be used to detect and identify *Psy* cultivation as an available and effective isolation method which can identify low concentrations of this pathovar from environmental infected samples [33]. The proficiency of the operator was used to identify and isolate the intended pathovar based on fluorescence and colony morphology. The colonies of *Psy* grew on King’s B medium. As Shaad’s work [1], after 48–72 h of incubation, fluorescence on King’s B medium was observed under UV light. Fifteen *Psy* pathovars (in 15 separate plates) from 50 samples were fluorescent on the KB medium (Fig. 8).

Since some other pathovars of *Pseudomonas syringae* also show positive responses to KB medium and produce green fluorescent pigment and, finally, lead to biases related to the use of phenotypic properties, it is not considered as a specific test to differentiate *Psy* from other pathovars of *Pseudomonas* [9, 10]. The LAMP technology is utilized in diagnostic laboratories for the rapid identification of several pathogenic bacteria in blood samples [34, 35]. However, no one has been employed in its application for the detection of *Psy*. The LAMP reaction itself takes place within 60 min, while only 3 h is required for perfect identification of cultivated cells [36]. The selection of *third* gene-based LAMP and PCR tests were reasonable because this gene should be conserved in all *Pseudomonas syringae* strains [25]. The presence of the *syrD* gene can be proved by PCR and LAMP tests specifically [37].

The specificity of the LAMP as the isothermal method in 60 °C and PCR test performed with an annealing temperature of 55 °C is shown in Fig. 2 and 3. In the LAMP section, lanes 2 and 3 indicate LAMP results of *Psy* with two pairs of primers. In the PCR section, lanes 4 and 5 show positive responses. Lane 3, as a negative control (*Xanthomonas ssp*). It means the related primer was specific for *Psy*. Both PCR and LAMP detection of the *syrD* gene were positive. Amplification of a unique DNA product in the *syrD* PCR showed the high specificity of the designed *syrD* gene primers. In 1999, Bultreys and Gheysen carried out a PCR test with designed primers for amplification of a 1040 bp fragment in the *syrD* gene coding sequence leading to efficient detection of the desired gene among related lipodepsipeptide-producing pathovars [18].

Guilbaud and colleagues in 2016 could efficiently perform isolation and identification of *Pseudomonas syringae* among the whole *P. syringae* group by using a method combining the PCR (named *Pseudomonas syringae*-specific polymerase chain reaction (Psy-PCR) detection and bacteria cultivation) [26]. Vincente et al. were discriminate *Pseudomonas syringae* isolates from sweet and wild cherry using rep-PCR [38]. Figure 4 contained the comparison of electrophoresis-based methods of PCR and LAMP products in terms of sensitivity. Similar dilution was prepared for both LAMP and PCR products. Lane 1 showed PCR product without dilution 10 (200 ng/μl), and lanes 7 and 8 showed LAMP product diluted by 10 (1 ng/μl) and 10^− 1^ (0.1 ng/μl), respectively.

The sensitivity of the lamp technique is 10 times higher than the PCR between these two electrophoresis-based methods. Figure 5 represented the results of gel-free and electrophoresis-based methods of PCR products in terms of sensitivity. In both parts, the same serial dilutions were prepared. In section A, only the first lane got a positive answer, but in section B, the positive answer showed on the third microtube.

The results indicated that the direct addition of SYBR Gold with PCR products in microtubes was 100 times more sensitive than electrophoresis in the direct visualization. Figure 6 contained the sensitivity of gel-free and gel-based staining of LAMP byproducts. In part A, lanes 2 and 3 showed the LAMP ladder-like band, and in part B, the positive answer showed in the third microtube. The results indicated that the direct addition of SYBR Gold with LAMP products in microtubes was 10 times more sensitive than electrophoresis in the direct visualization. All 15 colonies growing on 15 plates of KB medium gave positive in LAMP. One hundred percent of the Psy colonies on KB medium were detected by the LAMP technique. The specificity of LAMP primer pairs by testing on different kinds of stone fruits was shown in Fig. 7.

Based on Table 3, different results mean the LAMP technique outperforms the culturing method in terms of sensitivity and specificity. Despite reliability, specificity, and benefits of more speed, simplicity and sensitivity, in comparison with other methods [37, 39], in similar work (1998), Sorenson suggested that amplification of PCR with *syrD*-based primers, as revealed by cyclic lipodepsinonapeptide production or with southern blot analysis, did not always associate with the existence of the *syrD* gene [27, 40]. The comparison of Figs. 5 and 6 direct visualization shows that the sensitivity of loop-mediated isothermal amplification technique in detecting *Psy* is more than that of PCR. Moreover, the LAMP method is more rapid than PCR-based techniques, needs less time in comparison to PCR, and does not need any thermal cycler and expert staff.

PCR is used in numerous studies to identify pathogenic microorganisms. Amplification of *syrD* gene using PCR for identification of phytopathogenic strains of *P. syringae pv. syringae* has been already reported [4]. Kaluzna and colleagues studied characterization and genetic diversity of *Pseudomonas syringae* isolated from stone fruits and hazelnut using repetitive-PCR and MLST [21]. Gasic and colleagues could detect toxin-producing genes, *syrB*, and *syrD* in *Psy* within stone fruits by Rep-PCR [19]. LAMP primers have been reported to be able to detect other species of *Pseudomonas syringae pv. phaseolicola* [40].

Kumar Ghosh and colleagues using LAMP for the detection of *Candidatus liberibacter* in citrus and psyllid vector, *Diaphorina citri* Kuwayama asiaticus, report it as a good technique for early detection [41]. Keizerweerd et al. showed that LAMP and real-time PCR had the same sensitivity in 0.1 ng for the detection of *Puccinia kuehnii* and reported that LAMP was specific and rapid [42]. Herrera-Vasquez and colleagues used LAMP for the detection of Begomovirus species infecting tomato; they report the same sensitivity between LAMP and PCR, but mention that LAMP is a rapid specific and cheap method [43]. LAMP isothermal amplification has already been used to detect *Pseudomonas syringae pv. lachrymans* in cucumber leaves and was found to be a reliable and sensitive method [44]. LAMP assay showed to be a powerful tool for the detection of P. aeruginosa strains, as well [45]. Sun et al. were reported that the LAMP diagnostic assay contributes to the rapid and accurate detection of soft-rot disease in *Amorphophallus konjac* at an early stage [46]. LAMP-based detection showed to be more sensitive than PCR in detecting *Phytophthora hibernalis*, *P. syringae*, and *P. cambivora* [47]. The comparison of LAMP assays with direct bacterial cultivation showed high sensitivity and high specificity. When comparing the efficiency of the three mentioned techniques, LAMP was better than the PCR-based and culturing methods for its higher respective sensitivity and specificity. Hence, The LAMP test could work as a reliable and prompt tool to detect and identify with considerable applications in environmental and agricultural sciences. As shown in this study, *syrD* amplifying LAMP primers are efficient in isothermal gene amplification as well and can be used to detect Psy.

## Conclusions

Pathogen detection, identification, and quantification are important in plant disease control and must be accessible in all regions to ensure sustainable crop production and food safety to our knowledge. This study is the first to report on the comparison of different PCR-based assays culture and the LAMP technique for the detection of *Pseudomonas syringae pv. syringae* that particularly damaged stone fruits.

The current study described a novel molecular detection of *Pseudomonas syringae pv. syringae* that particularly damaged stone fruits. LAMP is a fast, highly specific and cheap tool for early molecular detection of Psy on stone fruits. The method does not need a thermal cycler; it will be practical for a larger number of researchers. LAMP techniques can eliminate biases for further classification and characterization of putative colonies.

## Data Availability

The datasets used and/(or) analyzed during the current study are available from the corresponding author on reasonable request.

## Abbreviations

B3: Backward outer primer
BIP: Backward inner primer
CFL: Corono facate ligase
F primer: Forward primer
F3: Forward outer primer
FIP: Forward inner primer
FN: False negatives
FP: False positives
GATTa: Gelatin liquefaction, aesculin hydrolysis, tyrosinase activity, tartrate utilization
KB: King’s B
LAMP: Loop-mediated isothermal amplification
LOD: Limit of detection
LOPAT: Levan production, oxidase production, pectinolytic activity, arginine dihydrolase production, tobacco hypersensibility
MLST: Multilocus sequence typing
Neg: Negative
PCR: Polymerase chain reaction
Pos: Positive
Psy: *Pseudomonas syringae pv. syringae*
R primer: Reverse primer
Rep-PCR: Repetitive polymerase chain reaction
syrB: Syringomycin B
syrD: Syringomycin D
TN: True negatives
TP: True positives
UV: Ultraviolet
